# Supplementary material for: EMC rectifies the topology of multipass membrane proteins
Source: Nat Struct Mol Biol. 2023 Nov 13;31(1):32–41. doi: 10.1038/s41594-023-01120-6 (PMC10803268; doi:10.1038/s41594-023-01120-6)

nature structural & molecular biology

Article

https://doi.org/10.1038/s41594-023-01120-6

EMC rectifies the topology of multipass membrane proteins

In the format provided by the authors and unedited

Supplemental Figure 1

Representative gating for
phycoerythrin (PE) labelled FLAG antibody

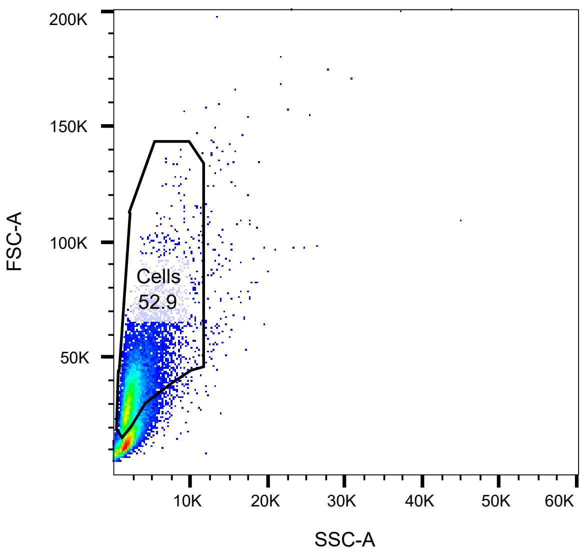

Representative gating for
GFP-P2A-RFP reporter constructs

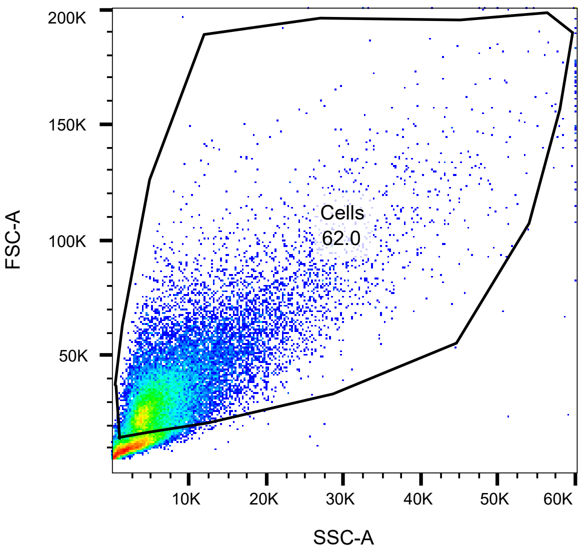

Supplement: Supplementary file 1 — Supplementary Fig. 1: Flow cytometry gating strategy. [file 41594_2023_1120_MOESM1_ESM.pdf]
