# Supplementary material for: EMC rectifies the topology of multipass membrane proteins
Source: Nat Struct Mol Biol. 2023 Nov 13;31(1):32–41. doi: 10.1038/s41594-023-01120-6 (PMC10803268; doi:10.1038/s41594-023-01120-6)
Supplement: Supplementary file 2 — Reporting Summary [file 41594_2023_1120_MOESM2_ESM.pdf]

nature portfolio

Corresponding author(s): Ramanujan Hegde
Last updated by author(s): Aug 26, 2023

Reporting Summary

Nature Portfolio wishes to improve the reproducibility of the work that we publish. This form provides structure for consistency and transparency in reporting. For further information on Nature Portfolio policies, see our Editorial Policies and the Editorial Policy Checklist.

Statistics

For all statistical analyses, confirm that the following items are present in the figure legend, table legend, main text, or Methods section.

n/a	Confirmed	
☐	☒	The exact sample size (*n*) for each experimental group/condition, given as a discrete number and unit of measurement
☐	☒	A statement on whether measurements were taken from distinct samples or whether the same sample was measured repeatedly
☒	☐	The statistical test(s) used AND whether they are one- or two-sided<br>*Only common tests should be described solely by name; describe more complex techniques in the Methods section.*
☒	☐	A description of all covariates tested
☒	☐	A description of any assumptions or corrections, such as tests of normality and adjustment for multiple comparisons
☐	☒	A full description of the statistical parameters including central tendency (e.g. means) or other basic estimates (e.g. regression coefficient) AND variation (e.g. standard deviation) or associated estimates of uncertainty (e.g. confidence intervals)
☒	☐	For null hypothesis testing, the test statistic (e.g. *F*, *t*, *r*) with confidence intervals, effect sizes, degrees of freedom and *P* value noted<br>*Give P values as exact values whenever suitable.*
☒	☐	For Bayesian analysis, information on the choice of priors and Markov chain Monte Carlo settings
☒	☐	For hierarchical and complex designs, identification of the appropriate level for tests and full reporting of outcomes
☒	☐	Estimates of effect sizes (e.g. Cohen's *d*, Pearson's *r*), indicating how they were calculated

Our web collection on statistics for biologists contains articles on many of the points above.

Software and code

Policy information about availability of computer code

Data collection	Phosphorimaging of radioactive samples was acquired on Typhoon FLA7000 (GE Healthcare). Flow cytometry data were collected using a Beckton Dickinson LSRII.
Data analysis	Fiji (Version 1.53c) was used to analyze band intensity in autoradiography. FlowJo (version 10.8.0) was used to analyze flow cytometry data.

For manuscripts utilizing custom algorithms or software that are central to the research but not yet described in published literature, software must be made available to editors and reviewers. We strongly encourage code deposition in a community repository (e.g. GitHub). See the Nature Portfolio guidelines for submitting code & software for further information.

Data

Policy information about availability of data

All manuscripts must include a data availability statement. This statement should provide the following information, where applicable:

- Accession codes, unique identifiers, or web links for publicly available datasets
- A description of any restrictions on data availability
- For clinical datasets or third party data, please ensure that the statement adheres to our policy

The paper contains all the data that was used to arrive at the conclusions of this study in main, extended and supplemental items. Publicly available datasets used in this study: Uniprot database (http://www.uniprot.org/), AlphaFold2 protein structure database (https://www.alphafold.ebi.ac.uk/).

Research involving human participants, their data, or biological material

Policy information about studies with human participants or human data. See also policy information about sex, gender (identity/presentation), and sexual orientation and race, ethnicity and racism.

Reporting on sex and gender	NA
Reporting on race, ethnicity, or other socially relevant groupings	NA
Population characteristics	NA
Recruitment	NA
Ethics oversight	NA

Note that full information on the approval of the study protocol must also be provided in the manuscript.

Field-specific reporting

Please select the one below that is the best fit for your research. If you are not sure, read the appropriate sections before making your selection.

☒ Life sciences ☐ Behavioural & social sciences ☐ Ecological, evolutionary & environmental sciences

For a reference copy of the document with all sections, see nature.com/documents/nr-reporting-summary-flat.pdf

Life sciences study design

All studies must disclose on these points even when the disclosure is negative.

Sample size	No sample size calculations were performed. Biochemical experiments and flow cytometry experiments were repeated on independent days to verify reproducibility. Flow cytometry measurements included a minimum of 30,000 live cells expressing the reporter. Each experiment was performed at least twice to verify that the same result was obtained in each case. The specific number of repeats for every experiment is included in the Methods section. The minimum sample size of two was chosen because extensive earlier published data on these types of bulk biochemical assays show that the type of processes we are studying displays very little variability from experiment to experiment. The number of cells chosen for analysis in flow cytometry was also based on prior published studies on this type of assay demonstrating that minimal variability is seen when 10,000 or more cells are analyzed.
Data exclusions	No data were excluded from the analysis.
Replication	Reproducibility and reliability of the findings has been ensured in several ways. In most cases, biochemical experiments in vitro and functional assays in cells were performed on separate and fully independent occasions and verified to give the same result as the example shown in the figure. All experiments are performed at least twice.
Randomization	Sample randomization is not relevant to our functional experiments because we routinely verify the identify of cells were used in our experiments. Hence, samples were not randomized for the functional assays because there is nothing to randomize. The different conditions being compared within any given experiment derive from a single common stock of reagent or a single culture of cells, so random assignment or covariates are not relevant to this type of study.
Blinding	Blinding is not performed in functional assays because the experiments have internal or external controls to indicate the identify of the cells we were assaying.

Reporting for specific materials, systems and methods

We require information from authors about some types of materials, experimental systems and methods used in many studies. Here, indicate whether each material, system or method listed is relevant to your study. If you are not sure if a list item applies to your research, read the appropriate section before selecting a response.

Materials & experimental systems

n/a	Involved in the study
☐	☒ Antibodies
☐	☒ Eukaryotic cell lines
☒	☐ Palaeontology and archaeology
☒	☐ Animals and other organisms
☒	☐ Clinical data
☒	☐ Dual use research of concern
☒	☐ Plants

Methods

n/a	Involved in the study
☒	☐ ChIP-seq
☐	☒ Flow cytometry
☒	☐ MRI-based neuroimaging

Antibodies

Antibodies used	CCDC47 (Bethyl Laboratories A305-100A, 1:5000); EMC3 (Invitrogen 711771, 1:5000); EMC6 (Abcam ab84902, 1:1000); Calnexin (Enzo ADI-SPA-865, 1:5000); Sec61α (ref70, 1:5000); TMCO1 (Invitrogen PA5-43350, 1:500); Sec61β (ref71, 1:5000); CAML (Cell Signaling Technology 13913S, 1:1000); FLAG M2-HRP (Sigma A8592, 1:5000); EMC4 (Abcam, ab123719, 1:2000); β-Actin-HRP (Sigma, A3854, 1:10000); phycoerythrin (PE) labelled FLAG antibody (BioLegend 637310, 1:100); GABRA1 antibody (Invitrogen, PA5-79291, 1.25μg per IP).
Validation	Each antibody was validated for specificity against the antigen by the manufacturer as follows:<br>phycoerythrin (PE) labelled anti-FLAG Tag Antibody: Validated for surface labeling by manufacturer (BioLegend). Species not applicable here because FLAG is an epitope tag that has no species.<br>Monoclonal ANTI-FLAG® M2-Peroxidase (HRP) antibody produced in mouse: validated by manufacturer (Sigma) for IB. Species not applicable here because FLAG is an epitope tag that has no species.<br>Anti-EMC4 antibody: validated for IB against human protein by manufacturer (Abcam) against human protein.<br>Anti-β-Actin−Peroxidase antibody, Mouse monoclonal: validated by manufacturer (Sigma) for IB against human protein.<br>GABRA1 antibody: validated by manufacturer (Invitrogen) for IB against human and mouse proteins.<br>CCDC47 antibody: validated by manufacturer (Bethyl Laboratories) for IB against human protein<br>EMC3 antibody: validated by manufacturer (Invitrogen) for IB against human protein.<br>EMC6 antibody: validated by manufacturer (Abcam) for IB against human protein.<br>Calnexin antibody: validated by manufacturer (Enzo) for IB against human protein.<br>TMCO1 antibody: validated by manufacturer (Invitrogen) for IB against human protein.<br>CAML antibody: validated by manufacturer (CST) for IB against human protein.<br>Sec61α: validated by previous work (Song et al., 2000) for IB against human protein.<br>Sec61β: validated by previous work (Fons et al., 2003) for IB against human protein.

Eukaryotic cell lines

Policy information about cell lines and Sex and Gender in Research

Cell line source(s)	HEK293 FRT/TO TRex cells were originally purchased from Invitrogen (R78007).<br>ΔEMC6 Flp-In™ T-REx™ 293 cells were described before (Guna et al., 2017) and were obtained from the Medical Research Council Laboratory of Molecular Biology (MRC-LMB).<br>ΔTMCO1 293 cell line was a gift from Robert Keenan (University of Chicago).<br>ΔEMC6ΔTMCO1 293 cell line was generated in this study.<br>Flp-In™ T-REx™ 293 cells stably expressing wild type or EMC3-FLAG mutants were described before (Wu and Hegde 2023) and were obtained from MRC-LMB.<br>Tetracycline-inducible HEK293 cell line expressing human GABAA receptor (alpha subunit is FLAG tagged at the N-terminus; beta subunit is untagged; gamma subunit is 1D4 tagged at the C-terminus) has been described before (Dostalova et al., 2014) and were obtained from MRC-LMB.<br>Flp-In™ T-REx™ 293 cell line stably expressing GFP-P2A-RFP-ASGR1 or GFP-P2A-RFP-SQS were described before (Chitwood et al., 2018) and were obtained from MRC-LMB.
Authentication	Cell lines were not authenticated beyond ensuring the presence of known antibiotic resistance markers within their genomes (by growth in the relevant antibiotics) and by their unique FRT site downstream of a doxycycline-inducible promoter as determined by the ability to integrate fluorescent reporters at this site.
Mycoplasma contamination	Cell lines were negative for mycoplasma. They are tested monthly.
Commonly misidentified lines<br>(See ICLAC register)	None used.

Flow Cytometry

Plots

Confirm that:

☒ The axis labels state the marker and fluorochrome used (e.g. CD4-FITC).

☒ The axis scales are clearly visible. Include numbers along axes only for bottom left plot of group (a 'group' is an analysis of identical markers).

☒ All plots are contour plots with outliers or pseudocolor plots.

☒ A numerical value for number of cells or percentage (with statistics) is provided.

Methodology

Sample preparation	Samples consisted of HEK293-derived cell lines that stably expressed a fluorescent protein reporter or GABAA receptor for monitoring surface expression. Where indicated in the Methods, they were first treated with siRNAs. The cells were collected in ice-cold PBS, washed and resuspended in PBS supplemented with 2% FCS and 1 µg/ml DAPI (Thermo Fisher Scientific). Where indicated, surface antibody/GABAA receptor labeling is performed after pelleting cells. Cells were passed through 70-µm filter immediately prior to analysis using Beckton Dickinson LSRII or LSRFortessa instrument. A total of at least 30,000 fluorescent and live (negative for DAPI stain) cells were collected.
Instrument	Beckton Dickinson LSRII or LSRFortessa.
Software	FlowJo (version 10.8.0).
Cell population abundance	A total of at least 30,000 live cells (negative for DAPI stain) that also were positive for the fluorescent protein reporter (either PE, GFP or RFP) were analyzed.
Gating strategy	Gating was used only to include cells, rather than debris. Further gating was not used.

☒ Tick this box to confirm that a figure exemplifying the gating strategy is provided in the Supplementary Information.
