# Supplementary figures and images for: EMC rectifies the topology of multipass membrane proteins
Source: Nat Struct Mol Biol. 2023 Nov 13;31(1):32–41. doi: 10.1038/s41594-023-01120-6 (PMC10803268; doi:10.1038/s41594-023-01120-6)

Figure 2

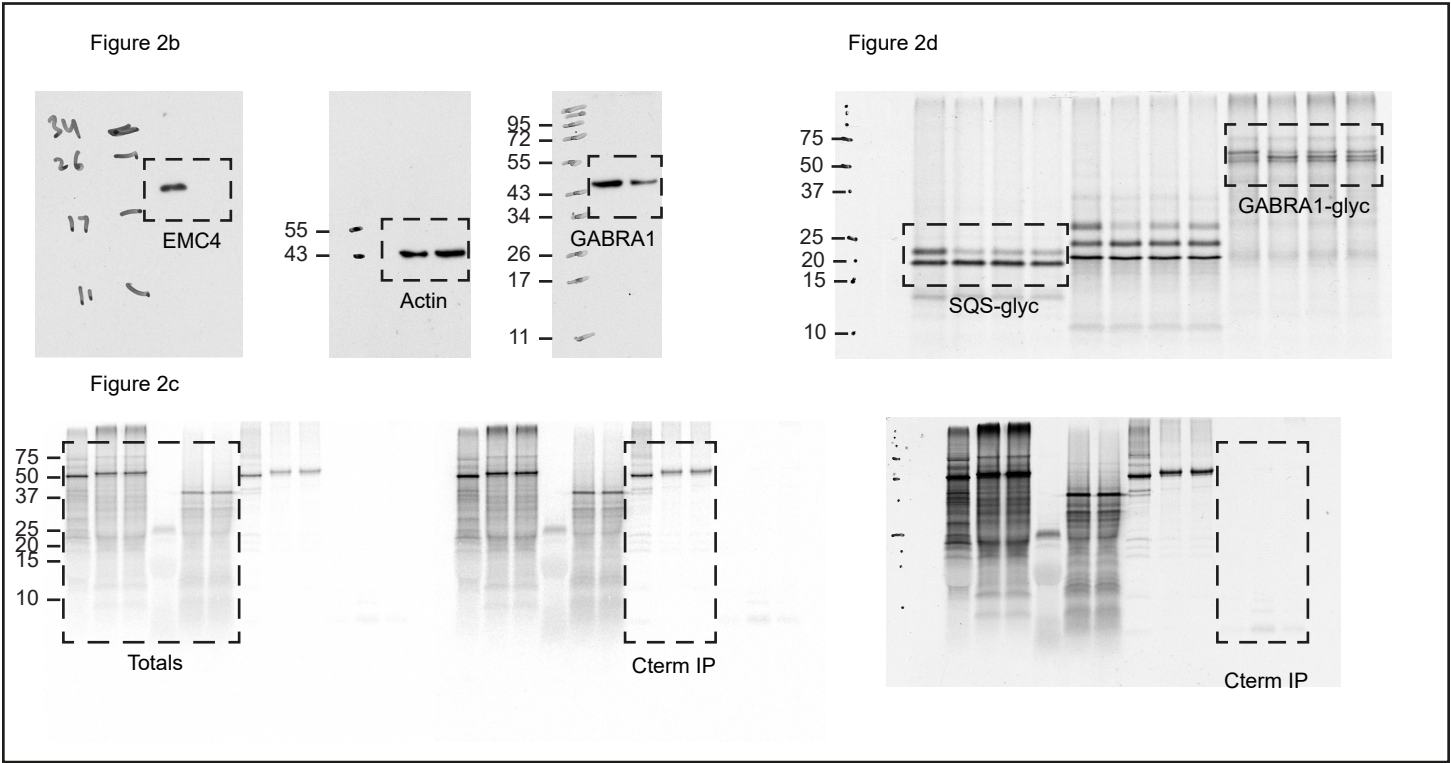

Supplement: Supplementary file 6 — Uncropped gels [file 41594_2023_1120_MOESM6_ESM.pdf]

Figure 3

Figure 3a

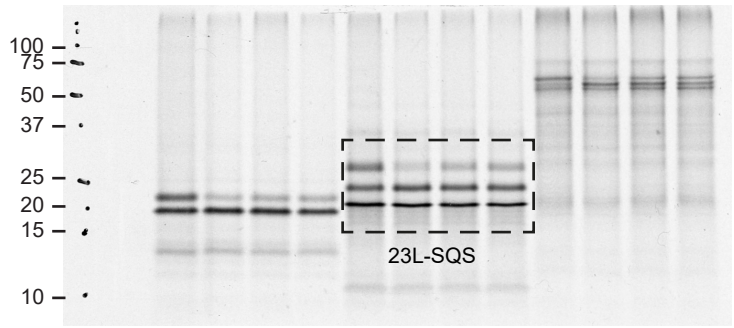

Figure 3b

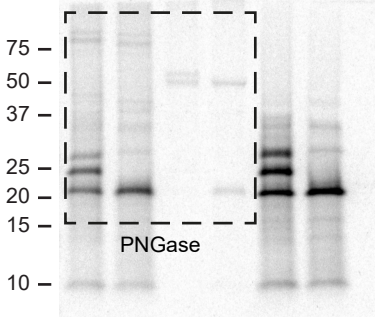

Figure 3b

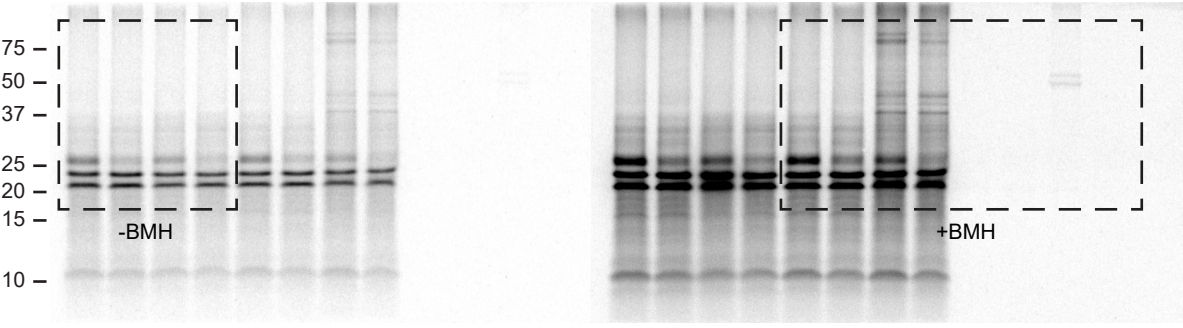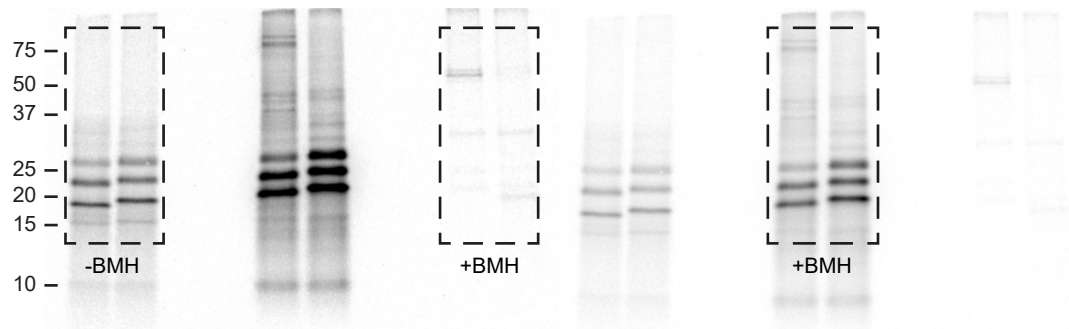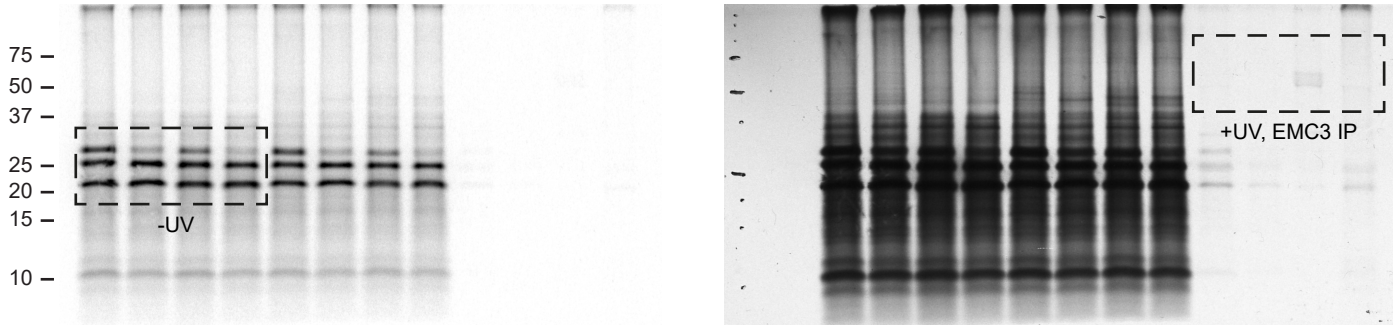

Supplement: Supplementary file 8 — Uncropped gels [file 41594_2023_1120_MOESM8_ESM.pdf]

Figure 4

Figure 4a

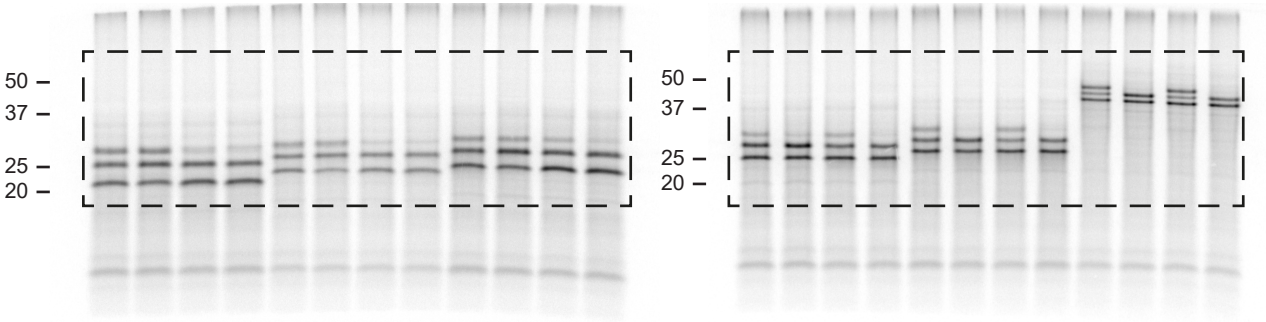

Figure 4b

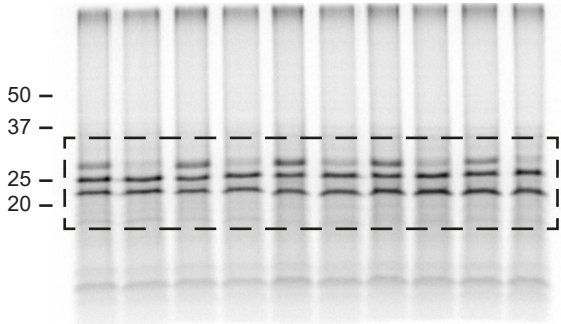

Figure 4c

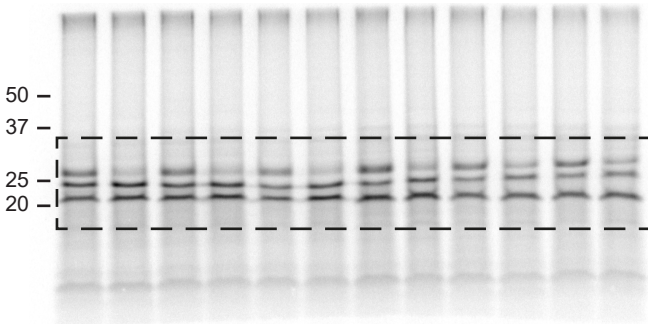

Supplement: Supplementary file 9 — Uncropped gels [file 41594_2023_1120_MOESM9_ESM.pdf]

Figure 5

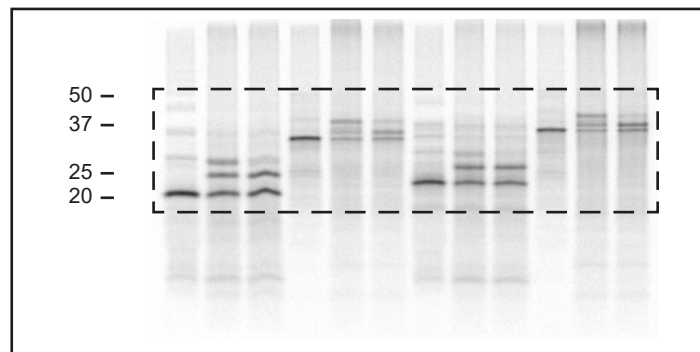

Supplement: Supplementary file 10 — Uncropped gels [file 41594_2023_1120_MOESM10_ESM.pdf]

Figure 6

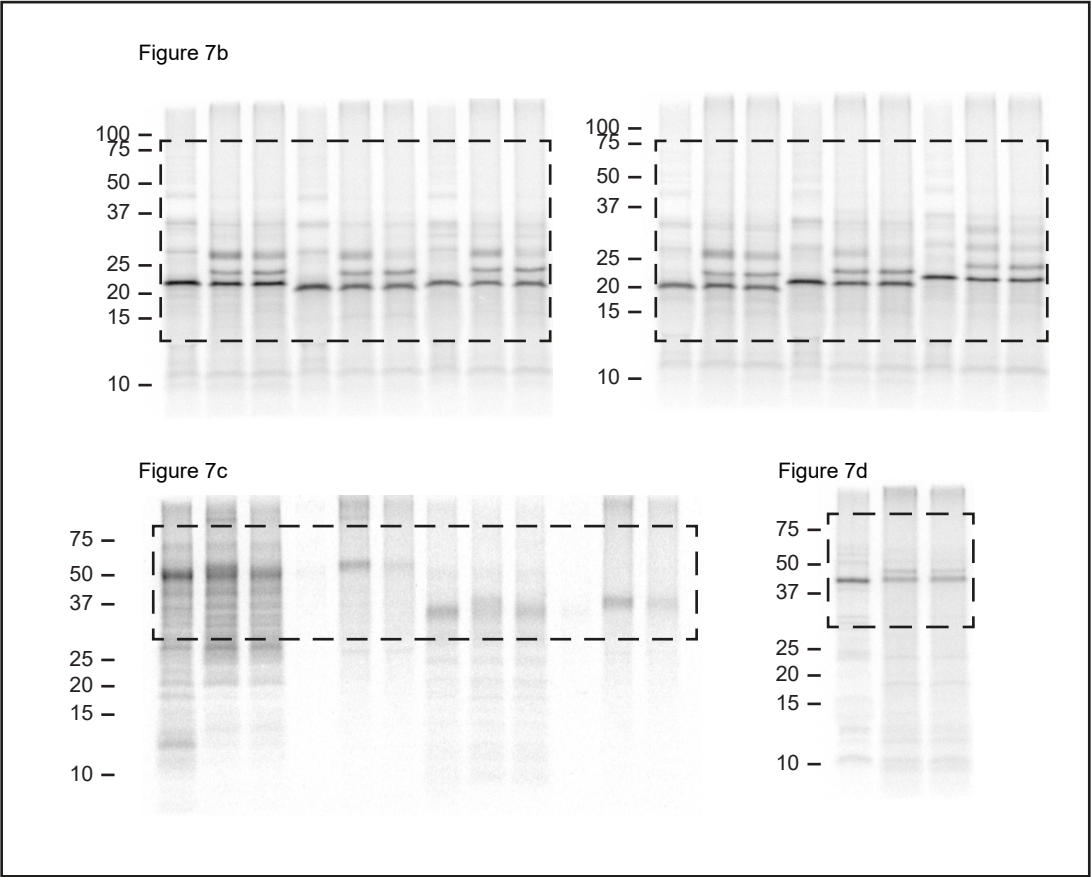

Supplement: Supplementary file 11 — Uncropped gels [file 41594_2023_1120_MOESM11_ESM.pdf]

Extended Data Figure 1

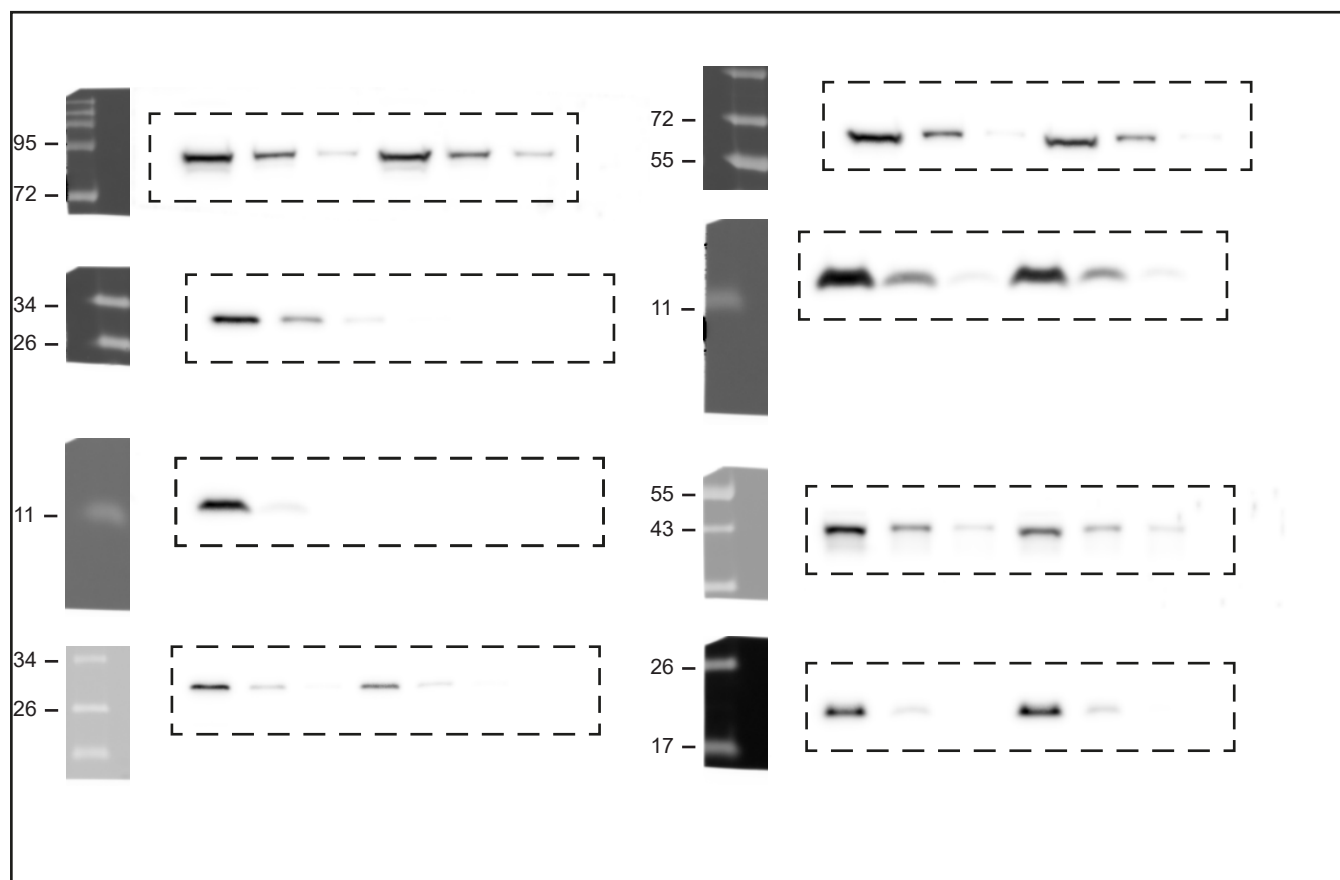

Supplement: Supplementary file 12 — Uncropped gels [file 41594_2023_1120_MOESM12_ESM.pdf]

Extended Data Figure 2

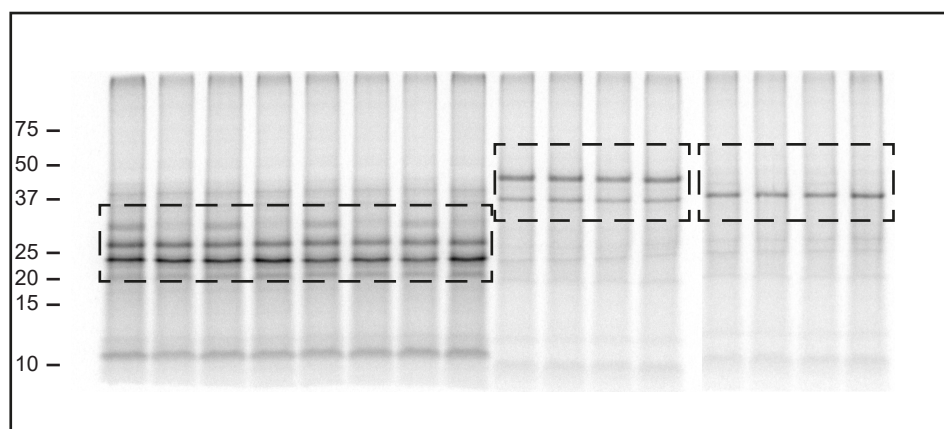

Supplement: Supplementary file 13 — Uncropped gels [file 41594_2023_1120_MOESM13_ESM.pdf]

Extended Data Figure 3

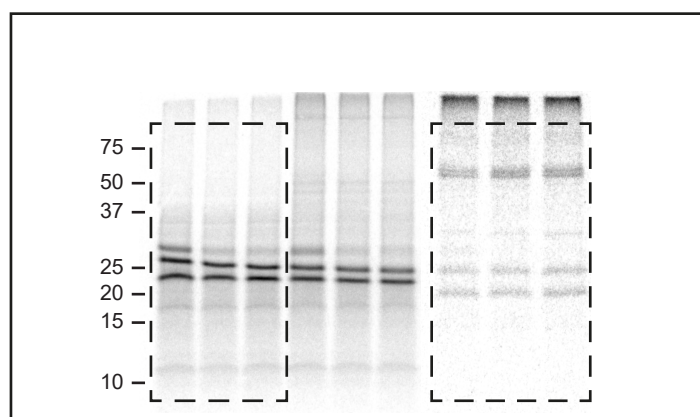

Supplement: Supplementary file 14 — Uncropped gels [file 41594_2023_1120_MOESM14_ESM.pdf]

Extended Data Figure 4

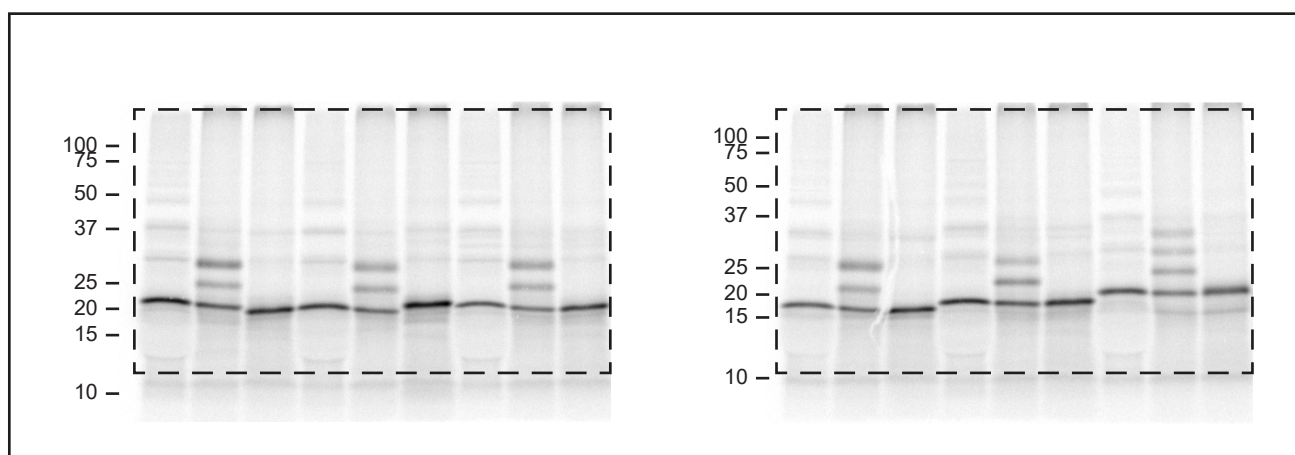

Supplement: Supplementary file 15 — Uncropped gels [file 41594_2023_1120_MOESM15_ESM.pdf]
